# Assessment of changes in cardiac volumes following MitraClip™ implantation using cardiac magnetic resonance imaging

**DOI:** 10.1186/1532-429X-14-S1-P101

**Published:** 2012-02-01

**Authors:** Ulf K Radunski, Maximilian Lange, Achim Barmeyer, Olaf Franzen, Volker Rudolph, Gunnar Lund, Gerhard Adam, Herrmann Reichenspurner, Stefan Blankenberg, Stephan Baldus, Kai Muellerleile

**Affiliations:** 1 Center for Cardiology and Cardiovascular Surgery, University Heart Center Hamburg, Hamburg, Germany; 2 Department of Diagnostic and Interventional Radiology, University Medical Center Hamburg-Eppendorf, Hamburg, Germany

## Summary

This study aimed at assessing left ventricular (LV), left atrial (LA) and right ventricular (RV) volumes in patients before and after MitraClip™ implantation by cardiac magnetic resonance imaging (CMR).

## Background

The MitraClip™ is a novel device for percutaneous mitral valve repair. Recent studies demonstrated a reduction of LV volumes after MitraClip™ implantation using echocardiography. CMR is currently the reference method to assess cardiac volumes but has not been used to assess LV remodeling after MitraClip™ implantation so far.

## Methods

Twelve patients with functional (n=7) or degenerative (n=5) mitral valve regurgitation grade 3 to 4 underwent CMR at baseline (BL) before and at 6 month follow-up (FU) after successful MitraClip™ implantation. CMR protocol consisted of short- and long-axis slices using a steady-state-free-precession cine sequence for the assessment of LV, LA and RV volumes.

## Results

**Figure 1:**
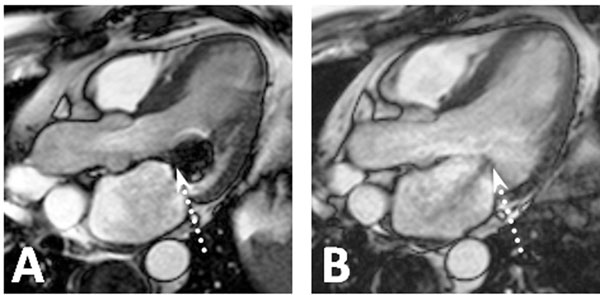


Minor device-related artifacts were observed, enabling reliable delineation of endocardial borders in all patients at FU. Figure 1) demonstrates typical device-related artifacts 6 month after implantation (A) in comparison with a corresponding pre-implantation image (B). Mean intra- and inter-observer biases were 0.9±2.0 and 1.6±2.9 % for LV end-diastolic (LVEDV), 0.3±4.7 and 1.8±6.4 % for LV end systolic (LVESV), 0.1±2.9 and 2.2±3.7 % for RVEDV, 1.7±7.8 and 3.5±8.8 % for RVESV as well as 0.3±7.6 and 13.7±14.0 % for LA (LAV) volume indices at FU. No significant differences in intra- or inter-observer biases were observed between BL and FU. LVEDV (127 (96-150) vs. 112 (86-150) ml/m^2^; p=0.03) as well as LVESV (82 (54-91) vs. 69 (48-99) ml/m^2^; p=0.03) indices significantly decreased from BL to FU. No significant difference was found for RVEDV (94 (75-103) vs. 99 (77-123) ml/m^2^; p=0.91), RVESV (48 (42-80) vs. 51 (40-81) ml/m^2^; p=0.48) and LAV (87 (55-124) vs. 92 (48-137) ml/m^2^; p=0.20) indices between BL and FU.

## Conclusions

CMR enables reproducible measurements of cardiac volumes in patients with implanted MitraClip™ devices. Significantly decreased LV but unchanged LA and RV volumes were found at 6 month after successful MitraClip™ implantation.

## Funding

No external funding.

